# Supplementary material for: A rapid transfer of virions coated with heparan sulfate from the ECM to CD151 defines an early step in the human papillomavirus infection cascade
Source: eLife. 2026 Jun 16;14:RP107139. doi: 10.7554/eLife.107139 (PMC13271742; doi:10.7554/eLife.107139)
Supplement: Peer review history

# Editor report: eLife Assessment

**DOI:** 10.7554/eLife.107139.4.sa0

**Authors:** John W Schoggins

**Affiliations:** The University of Texas Southwestern Medical Center United States

**Keywords:** Convincing, Valuable

This study provides **valuable** insight into the role of actin protrusions in mediating early pre-endoyctic steps of human papillomavirus entry at the cell surface. Using state-of-the-art microscopy in an immortalized keratinocyte model, the authors present **convincing** evidence that filopodia actively promote the transfer of heparin sulfate-coated virions from the extracullar matrix to the viral entry factor CD151. These findings provide a strong framework for future studies aimed at further resolving the dynamics of virion transfer and receptor engagement.

# Referee report: Reviewer #1 (Public review)

**DOI:** 10.7554/eLife.107139.4.sa1

[Editors' note: this version has been assessed by the Reviewing Editor without further input from the original reviewers. The editors have determined that the authors adequately addressed the prior reviewer comments.]

Summary:

The author's goal was to arrest PsV capsids on the extracellular matrix using cytochalasin D. The cohort was then released and interaction with the cell surface, specifically with CD151 was assessed.

Note on previous revisions:

The authors did an excellent job in their revision to include data from the effect of proteolytic priming on their observed virion transfer to the cell body. All other minor issues were addressed adequately.

The work could be especially critical to understanding the process of in vivo infection.

# Referee report: Reviewer #2 (Public review)

**DOI:** 10.7554/eLife.107139.4.sa2

Review of the previous version:

The study design involves infecting HaCaT cells (immortalised keratinocytes mimicking basal cells of a target tissue) and observing virus localization with and without actin polymerization inhibition by cytochalasin D (cytoD) to analyze virion transfer from the ECM to the cell via filopodial structures, using cellular proteins as markers.

In the context of the model system, the authors stress in the revised version the importance of using HaCaT cells as a relevant 'polarized' cell model for infection. The term 'polarized' is used in the cell biological literature for epithelial cells to describe a strict apical vs. basolateral demarcation of the plasma membrane with an established diffusion barrier of the tight junction. However, HaCat cells do not form tight junctions. In squamous epithelia, such barriers are only found in granular layers of the epithelium. The published work cited in support of their claims either does not refer to polarity or only in the context of other cells such as CaCo-2 cells.

Overall, the matter of polarity would be important, if indeed the virus could only access cell-associated HSPGs as primary binding receptor, or the elusive secondary receptor via the ECM in the used model system (HaCaT cells), if they would locate exclusively basolaterally. This is at least not the case for binding, as observed in several previous publications (just two examples: Becker et al, 2018, Smith et al., 2008). With only a rather weak attempt at experimental verification of their model system with regards to polarity of binding, the authors then go on to base their conclusions on this unverified assumption.

This is one example of several in the manuscript, where claims for foundational premises, observations, and/or conclusions remain undocumented or not supported by experimental data.

Another such example is the assumption of transfer of the virus from ECM to the tetraspanin CD151. Here, the conclusions are based on the poorly documented inability of the virus to bind to the cell body, which is in stark contrast to several previous publications, and raises questions. Thus, association with CD151 likely occurs both from ECM derived virus AND virus that binds to cells, so that any conclusions on the mode of association is possible only in live cell data (which is not provided). Overall, their proposed model thus remains largely unsubstantiated with regards to receptor switching.

There are a number of important additional issues with the manuscript:

First, none of the inhibitors have been tested in their system for efficacy and specificity, but rely on published work in other cell types. This considerably weakens the confidence on the conclusion drawn by the authors.

Second, the authors aim to study transfer from ECM to the cell body and effects thereof. However, there are still substantial amounts of viruses that bind to the cell body compared to ECM-bound viruses in close vicinity to the cells. This is in part obscured by the small subcellular regions of interest that are imaged by STED microscopy, or by the use of plasma membrane sheets. This remains an issue despite the added Supple. Fig. 1, where also only sub cellular regions are being displayed. As a consequence the obtained data from time point experiments is skewed, and remains for the most part unconvincing, largely because the origin of virions in time and space cannot be taken into account. This is particularly important when interpreting the association with HS, the tetraspanin CD151, and integral alpha 6, as the low degree of association could be originating from cell bound and ECM-transferred virions alike.

Third, the use of fixed images in a time course series also does not allow to understand the issue of a potential contribution of cell membrane retraction upon cytoD treatment due to destabilisation of cortical actin. Or, of cell spreading upon cytoD washout. The microscopic analysis uses an extension of a plasma membrane stain as marker for ECM bound virions, this may introduce a bias and skew the analysis.

Fourth, while the use of randomisation during image analysis is highly recommended to establish significance (flipping), it should be done using only ROIs that have a similar density of objects for which correlations are being established. For instance, if one flips an image with half of the image showing the cell body, and half of the image ECM, it is clear that association with cell membrane structures will only be significant in the original. But given the high density of objects on the plasma membrane, I am not convinced that doing the same by flipping only the plasma membrane will not also obtain similar numbers than the original.

# Author comment: Author response

**DOI:** 10.7554/eLife.107139.4.sa3

**Authors:** Annika Massenberg, Yahya Homsi, Carl Niklas Schneider, Snježana Mikuličić, Tatjana Döring, Luise Florin, Thorsten Lang

**Affiliations:** University of Bonn Bonn Germany; University of Bonn Bonn Germany; University of Bonn Bonn Germany; Johannes Gutenberg University Mainz Mainz Germany; Johannes Gutenberg University Mainz Mainz Germany; University Medical Center of the Johannes Gutenberg University Mainz Mainz Germany; University of Bonn Bonn Germany

The following is the authors’ response to the previous reviews

**eLife Assessment**

This study provides valuable insight into the role of actin protrusions in mediating early pre-endoyctic steps of human papillomavirus entry at the cell surface. Using state-of-the-art microscopy in an immortalized keratinocyte model, the authors present mostly solid evidence that filopodia actively promote the transfer of heparin sulfate-coated virions from the extracullar matrix to the viral entry factor CD151. Remaining gaps in the mechanistic model could be further supported by including a more expansive analysis of the fixed microscopy samples and live cell imaging to distinguish virion transfer from direct binding.

We thank the editorial team for the improved eLife assessment. Regarding the remaining gap, we agree that it is not clear why the large majority of the virions indeed are transferred and not directly binding virions.

**Public Reviews:**

**Reviewer #1 (Public review):**

Summary:

The author's goal was to arrest PsV capsids on the extracellular matrix using cytochalasin D. The cohort was then released and interaction with the cell surface, specifically with CD151 was assessed.

The model that fragmented HS associated with released virions mediates the dominant mechanism of infectious entry has only been suggested by research from a single laboratory and has not been verified in the 10+ years since publication. The authors are basing this study on the assumption that this model is correct, and these data are referred to repeatedly as the accepted model despite much evidence to the contrary. The discussion in lines 65-71 concerning virion and HSPG affinity changes is greatly simplified. The structural changes in the capsid induced by HS interaction and the role of this priming for KLK8 and furin cleavage has been well researched. Multiple laboratories have independently documented this. If this study aims to verify the shedding model, additional data needs to be provided.

Comment of the authors: the above paragraph is copied from the very first review and describes the situation before revision.

Note on revisions:

The authors did an excellent job in their revision to include data from the effect of proteolytic priming on their observed virion transfer to the cell body. All other minor issues were addressed adequately.

We are grateful that the referee acknowledges that we addressed all issues adequately.

The work could be especially critical to understanding the process of in vivo infection.

We agree, and would like to point out that a similar comment was raised by the reviewing editor assigned to our original submission, John Schiller. For unknown reasons, he was no longer involved in the evaluation of the revision.

**Reviewer #2 (Public review):**

The study design involves infecting HaCaT cells (immortalised keratinocytes mimicking basal cells of a target tissue) and observing virus localization with and without actin polymerization inhibition by cytochalasin D (cytoD) to analyze virion transfer from the ECM to the cell via filopodial structures, using cellular proteins as markers.

In the context of the model system, the authors stress in the revised version the importance of using HaCaT cells as a relevant 'polarized' cell model for infection. The term 'polarized' is used in the cell biological literature for epithelial cells to describe a strict apical vs. basolateral demarcation of the plasma membrane with an established diffusion barrier of the tight junction. However, HaCat cells do not form tight junctions. In squamous epithelia, such barriers are only found in granular layers of the epithelium. The published work cited in support of their claims either does not refer to polarity or only in the context of other cells such as CaCo-2 cells.

We thank the reviewer for this important clarification and fully agree. HaCaT cells do not form tight junctions and therefore do not fulfill the classical definition of polarized epithelial cells with a strict apical basolateral diffusion barrier. In response to this comment, we have removed the term “polarized” in reference to HaCaT cells throughout the revised manuscript. Our intention was not to imply classical epithelial polarity, but rather to emphasize that HaCaT cells represent a functionally relevant keratinocyte model that recapitulates key early steps of HPV infection observed in vivo, particularly abundant ECM deposition enabling for strong virion binding to the ECM.

We now state on line 120: “PsVs that bind to the ECM at sites distal from the cell body are unable to establish direct contact with entry receptors, until the cell migrates onto them or they are transported along cell protrusions towards the cell body (Schelhaas et al., 2008; Smith et al., 2008). Both cell migration and protrusion transport depend on actin dynamics (Schaks et al., 2019). We aimed for blocking these active recruitment mechanisms in HaCaT cells, a cell line that is widely used as a cell culture model for HPV infection. They resemble primary keratinocytes in several key aspects: they are not virally transformed and produce large amounts of ECM, promoting interactions between viruses and ECM components and thereby facilitating infection (Bienkowska-Haba et al., 2018; Gilson et al., 2020). In addition, subconfluent HaCaT cells form filopodia and filopodial transport is used for the recruitment of ECM-bound virus particles to the cell body (Schelhaas et al., 2008, Smith et al., 2008). Together, these features make HaCaT cells a suitable model for studying active PsV recruitment from the ECM to the cell surface.”

Overall, the matter of polarity would be important, if indeed the virus could only access cell-associated HSPGs as primary binding receptor, or the elusive secondary receptor via the ECM in the used model system (HaCaT cells), if they would locate exclusively basolaterally.

We apologize for not having stressed enough that virions bind as well directly to the not imaged, upper cell membrane. To make clear that HaCaT cells are still a suitable model for studying active recruitment, throughout the manuscript, we worked on the following issues (this is an outline, for details see below):

(1) We now discuss adequately that virions reach cell surface receptors either by passive diffusion or by active transport mechanisms, the latter involving actin dynamics (filopodial transport and cell migration), to which we refer in the revised manuscript as active recruitment.

(2) We explain why the large majority of virions in the microscopic assay are actively recruited virions.

(3) We explain the difference between biochemical infection assays that do not differentiate between passive and active recruitment, and microscopic assays studying the basal cell membrane and by this primarily actively recruited virions

This is at least not the case for binding, as observed in several previous publications (just two examples: Becker et al, 2018, Smith et al., 2008). With only a rather weak attempt at experimental verification of their model system with regards to polarity of binding, the authors then go on to base their conclusions on this unverified assumption.

We agree with the reviewer that strict epithelial polarity would only be relevant if HPV binding or receptor accessibility were confined to the basolateral membrane, which is not the case in HaCaT cells, as shown previously (e.g., Becker et al., 2018; Smith et al., 2008). However, our conclusions do not rely on strictly polarity-dependent binding.

We added the following paragraphs clarifying that (i) in HaCaT cells PsVs also bind by passive diffusion to the upper cell membrane and that (ii) at the basal membrane the large majority of imaged PsVs is actively recruited.

Line 332: “…, the lower PCC at 0 min/CytD suggests that without active recruitment less PsVs reach CD151. At 30 min after CytD, the PCC has reached the level of 0.1 as in the control, which is in line with the idea of fast recruitment as observed in Figure 4. To follow how the basal cell membrane is populated with PsVs over time, as additional analysis we determined the PsVs per µm^2^ in ROIs placed in the cell body region. At 0 min, CytD reduces the PsV density to 19 - 33%, albeit the effect is not significant, and at 180 min/CytD the same PsV density as in the control is reached (Supplementary Figure 6A and B). Overall, under CytD there was a trend towards less PsVs present (Supplementary Figure 6A and B). Hence, both Figure 5C and Supplementary Figure 6A and B suggest that active virion transport is required to reach efficiently the basal membrane.”

Line 447: “Throughout all experiments, we observe at 0 min/CytD only few PsVs at the basal membrane (Figure 1A, Supplementary Figure 6A and B; see also PCC at 0 min between PsVs an CD151 in Figure 5C), suggesting that in the absence of active recruitment the access to the basal membrane via passive diffusion is limited. We wondered, how many PsVs may bind to the cell membrane without a diffusion barrier? For this reason, we incubated EDTA detached HaCaT cells in suspension with PsVs for 1 h at 4 °C, followed by re-attachment for 1 h. Under these conditions, we find, despite of a shorter incubation time (1 h versus 5 h), a roughly 3-fold larger PsV density (1.7 PsVs/µm^2^ (Supplementary Figure 6D)) than the highest density observed in the other experiments. However, it should be noted that values of the different experiments cannot be directly compared. Aside from the different treatments, another difference lies in the size of the imaged membrane. The re-attachment of cells is not complete after 1 h (compare size of adhered membranes in Supplementary Figure 6A and 1A), wherefore the membranes are likely strongly ruffled, which results in the underestimation of the membrane area. As a result, we overestimate the PsVs per µm^2^ adhered membrane (please note that we cannot re-attach cells for longer times as we then lose PsVs due to endocytosis). In any case, the experiment suggests that PsVs bind more efficiently to membrane surface receptors without a diffusion barrier. We conclude that in our assay PsVs cannot readily bypass the active PsV recruitment by diffusing directly to the basal cell membrane, which is plausible, because to make this happen a 55 nm large PsV must diffuse through the narrow gap between glass-coverslip and adhered cell.”

Line 538: “The analyzed PsVs hardly bind to the basal cell surface directly by diffusion (Supplementary Figure 6, compare PsV maxima density at 0 min/CytD in A and B to C). Therefore, the actin-driven virion transport would play a decisive role in HPV infection if cells would form a monolayer with a disruption at which ECM is present and that is approached by PsVs, a scenario similar to in vivo infection. In addition, cell migration could establish contact between PsVs and the cell surface.”

Line 548: “…that can readily bind to the upper cell membrane. We are not aware of a PsV translocation mechanism from the upper to the basal membrane. Therefore, in our assay, PsVs bound to the upper membrane are not expected to show up at the basal membrane. Comparing 0 min of control and CytD (Supplementary Figure 6A and B), we find that compared to the control 19 - 33% of the PsVs reach the basal membrane in the absence of active transport, or in other words, most likely by passive diffusion. Actually, the range from 19 – 33% must be a strong overestimate as PsVs in the control are in transit and many actively recruited PsVs are already internalized during the 5 h incubation period. For this reason, we propose that most likely much less than 10% of the PsVs reach the basal membrane by diffusion. Moreover, in the absence of the diffusion barrier, the density of bound PsVs is strongly increased (Supplementary Figure 6D), showing indirectly that at the basal membrane the binding sites are difficult to access without active recruitment. Taken together, we propose the large majority of PsVs analyzed in our assay are ECM bound and actively recruited to the basal cell membrane.”

This is one example of several in the manuscript, where claims for foundational premises, observations, and/or conclusions remain undocumented or not supported by experimental data.

Another such example is the assumption of transfer of the virus from ECM to the tetraspanin CD151. Here, the conclusions are based on the poorly documented inability of the virus to bind to the cell body, which is in stark contrast to several previous publications, and raises questions.

We hope with the above changes we made clear that virions can also directly bind to the cell body. We also added a paragraph discussing differences between biochemical and microscopic assays.

Line 568: “In this scenario, sub-confluent HaCaT cells, or even better single HaCaT cells, would be an ideal model system for the microscopic study of these very early infection steps that involve ECM attachment and subsequent active recruitment, as supposed to occur during in vivo infection of basal keratinocytes after binding of virions to the basement membrane (Bienkowska-Haba et al., 2018; Day and Schelhaas, 2014; Kines et al., 2009; Schiller et al., 2010). In contrast, in biochemical infection assays, virions diffusing to HSPGs on the cell surface, and by this bypassing active recruitment, are assayed together with the actively recruited virions. Should cells secrete little ECM and are grown to confluency, the passively binding virions are supposed to strongly dominate the infection rate in a biochemical infection assay.”

There are a number of important additional issues with the manuscript:

First, none of the inhibitors have been tested in their system for efficacy and specificity, but rely on published work in other cell types. This considerably weakens the confidence on the conclusion drawn by the authors.

We use inhibitors CytD, blebbistatin, leupeptin and furin inhibitor I. The below references are examples reporting the usage of the inhibitors on HaCaT cells studied in the context of HPV infection.

Furin inhibitor I:

Cruz et al., Cleavage of the HPV16 Minor Capsid Protein L2 during Virion Morphogenesis Ablates the Requirement for Cellular Furin during De Novo Infection. Viruses, 2015; doi.org/10.3390/v7112910

Cytochalasin D/Blebbistatin:

Schelhaas et al., Human papillomavirus type 16 entry: retrograde cell surface transport along actinrich protrusions. PLoS Pathog., 2008. doi: 10.1371/journal.ppat.1000148.

Smith et al., Virus activated filopodia promote human papillomavirus type 31 uptake from the extracellular matrix. Virology, 2009; doi.org/10.1016/j.virol.2008.08.040 and

Leupeptin/Furin inhibitor I:

Cerqueira et al., Kallikrein-8 Proteolytically Processes Human Papillomaviruses in the Extracellular Space To Facilitate Entry into Host Cells. J. Virology, 2015; doi.org/10.1128/jvi.00234-15

Moreover, the reversible inhibitory effect of CytD the key inhibitor, used in this study on transport and infection is validated in this study. However, we discuss this data now in the context of directly binding virions more critically.

Line 485: “Hence, the infection assay suggests that the treatment is largely reversible and only slightly harmful, if at all. However, the luciferase infection assay does not distinguish between actively recruited PsVs and PsVs that bind passively by diffusion to the upper membrane. The latter fraction likely dominates the total infection rate and should be less affected by CytD than the fraction of actively recruited PsVs. Therefore, if the infection pathway of a small fraction of actively recruited PsVs is irreversibly inhibited, we may not be able to detect this effect on the background of unaffected passively binding PsV.”

Second, the authors aim to study transfer from ECM to the cell body and effects thereof. However, there are still substantial amounts of viruses that bind to the cell body compared to ECM-bound viruses in close vicinity to the cells.

Regarding direct binding to the cell body, please see our detailed reply above.

This is in part obscured by the small subcellular regions of interest that are imaged by STED microscopy, or by the use of plasma membrane sheets. This remains an issue despite the added Supple. Fig. 1, where also only sub cellular regions are being displayed. As a consequence the obtained data from time point experiments is skewed, and remains for the most part unconvincing, largely because the origin of virions in time and space cannot be taken into account. This is particularly important when interpreting the association with HS, the tetraspanin CD151, and integral alpha 6, as the low degree of association could be originating from cell bound and ECM-transferred virions alike.

We hope with the above explanations it is plausible that the imaged virions primarily reach the basal membrane by active recruitment.

Third, the use of fixed images in a time course series also does not allow to understand the issue of a potential contribution of cell membrane retraction upon cytoD treatment due to destabilisation of cortical actin. Or, of cell spreading upon cytoD washout. The microscopic analysis uses an extension of a plasma membrane stain as marker for ECM bound virions, this may introduce a bias and skew the analysis.

The referee is correct in pointing out that cell spreading after CytD wash off would affect our analysis, e.g. by increasing the overlap between PsVs and the cell body although no active recruitment via filopodial transport and cell migration occurs. An argument speaking against this possibility is the lack of increase in the PCC between PsVs and F-actin after CytD removal, if the protease inhibitor leupeptin was present (Figure 2B and D). Leupeptin prevents PsV/phalloidin overlap despite restored actin polymerization after washout of both inhibitors, suggesting that priming is required for increased PsV–actin association and is too slow to change PCC within 60 min. These results support that the observed overlap reflects active, priming-dependent recruitment rather than cell morphology changes.

We state on line 252: “Moreover, the experiment suggests that without PsV priming the PCC between PsV-L1 and F-actin does not increase, for instance, due to cell spreading after CytD removal.”

On line 494, we state “However, we assume that this is rather unlikely, as cell spreading would increase the PCC between PsVs and F-actin under a condition where PsVs are not-primed (and therefore not actively recruited) but cell spreading occurs, which is not the case in Figure 2B and D (CytD/leupeptin).”

Fourth, while the use of randomisation during image analysis is highly recommended to establish significance (flipping), it should be done using only ROIs that have a similar density of objects for which correlations are being established. For instance, if one flips an image with half of the image showing the cell body, and half of the image ECM, it is clear that association with cell membrane structures will only be significant in the original. But given the high density of objects on the plasma membrane, I am not convinced that doing the same by flipping only the plasma membrane will not also obtain similar numbers than the original.

Regarding the association of PsVs with CD151 and HS, we corrected for random background with reference to a calibration line that describes the random background association in dependence of the density of objects. We now refer to this issue on line 343: “…, the fraction of PsVs closely associated with CD151 is around 10% (Figure 5D, control), after correction for random background association, for which we used a calibration line based on the same density of PsVs in flipped images (see Supplementary Figure 7).”

In the legend of Supplementary Figure 7 we state: “…The fraction of closely associated PsVs (PsV-L1 maxima with a distance ≤ 80 nm to the next nearest CD151 maximum) in the Control of Figure 5 was analyzed on original and flipped images (for an example of a flipped image see Supplementary Figure 5A)…on flipped images, we often find values more than half of the values of the original images, demonstrating that many PsVs have a distance ≤ 80 nm to CD151 merely by chance, in the following referred to as background association…We take the altogether 24 fraction values obtained on flipped images (12 values from Control and CytD each), and plot the fraction of closely associated PsVs against the average CD151 maxima density in the respective images. As can be seen in (C), the fraction increases with the maxima density, as the chance of a distance ≤ 80 nm increases with the maxima density. The fitted linear regression line describes how the background association depends from the maxima density. As a result, the background association (y) can be calculated for any maxima density (x) with the equation y = 2.04 • x. The CytD/0 min condition may be overcorrected, if it includes many images with CD151 flipped onto peripheral PsVs that actually are distal to CD151 (for an example ROI see Supplementary Figure 5A). On the other hand, PsVs right at the cell border, where CD151 staining tends to be strong (Supplementary Figure 5A), after flipping have less CD151 than before, contributing to undercorrection.”

When omitting the CytD/0 min values, we obtain essentially the same calibration line.

**Recommendations for the authors:**

**Reviewer #2 (Recommendations for the authors):**

There are further issues that are not pertaining to the study design that I find important.

Fig.1

There are few, if any, filopodia in untreated cells. It would be good to quantify their abundance to substantiate that resting HaCat cells are indeed a good model for filopodial transport bs. membrane retraction / spreading.

We see filopodia in untreated HaCaT cells (although quite variable in abundance, please see control cells in e.g. Figure 3 and 8 and Supplementary Figure 2).

In HaCat ECM the virus binds also to laminin-332 for a good part. Would this not also confound the analysis?

We agree with the reviewer that in HaCaT-derived ECM, virus binding is not restricted to heparan sulfate (HS), and that laminin-332 represents an additional relevant binding partner. Indeed, viruses bound to laminin-332 may likewise be transported toward the cell body via laminin-binding integrins. We therefore consider laminin-332 to act as a parallel attachment factor alongside HS rather than as a mutually exclusive alternative.

However, the primary aim of this study was not to comprehensively map all ECM binding partners, but to analyze the actin-dependent transport of ECM-bound virus particles. HS was chosen as a representative and well-characterized ECM marker for initial virus attachment. Importantly, inhibition of actin dynamics by cytochalasin D blocks this transport process downstream of initial binding. Thus, irrespective of whether the virus is initially bound to HS, laminin-332, or both, the readout reflects interference with the same actin-dependent transport mechanism.

Consequently, the presence of laminin-332 binding does not confound our analysis, as the experimental outcome is determined by inhibition of transport rather than by the specific ECM attachment factor. Nonetheless, we acknowledge laminin-332 as an important parallel interaction partner and had already mentioned it the first version of the manuscript, but removed the sentence during the last revision, that has now been added again. On line 593 we state: “Finally, not all PsVs bound to the ECM are expected to bind to HS but could also bind to laminin-332 (Culp et al., 2006).”

Fig.2

Would benefit from live cell analysis. There are considerable amounts of virions on the cell body, which partially contradicts statements from Fig. 1. The fast transfer to the cell body after cyto D washout is based on the assumption that filopodia formation and transport along them (and not membrane extension) occurs quickly. Is this reasonable? Does membrane extension and migration occur between 0 min and later time points?

Regarding membrane extension after CytD removal, that in the analysis may be indistinguishable from active recruitment transfer, please see our reply above (no PCC increase between PsV-L1 and F-actin after CytD removal if leupeptin is employed). Regarding migration, we now included this possibility as an active recruitment mechanism that may occur in parallel to filopodial transport (please see our reply above).

Fig.4

How are the subcellular ROIs chosen? Is there not a bias by not studying a full cell?

In Figure 4 we are specifically interested in the time course of PsV diminishment from the cell periphery. The ROIs are generated with reference to the membrane staining, using the cell body delineation as a starting point. For details about how ROIs are generated, please see legend of Figure 4 and materials and methods.

Fig. 5/6

The data needs a better analysis on correlation by using randomisation as explained above.

Please see our reply above. The association between PsVs and CD151 or HS has been corrected using a calibration line based on the same density of objects.

Fig. 8. Why does blebbistatin block the transport only partially? Previous work on actin retrograde flow suggests that in the absence of myosin II function the transport stops completely. Would this not be a concern, when interpreting the city D data?

Is the referee referring to Schelhaas et al., 2008 that we cite in the paper? In this paper, in HeLa cells blebbistatin reduced the directed particle motion by 82%, but not completely.

Suppl. Fig. 1A, B: Intented to adress the issue of viruses binding to the cell body, it unfortunately falls short. It would have been better to analyse complete cells rather than ROIs, or better even, a comprehensive analysis of cell islets (boundary cells vs. central cells, with cell body to cell periphery).

This experiment addresses the increase in PsV density resulting from active recruitment. Outlining entire cells would include also PsVs close to the cell edge that have not been actively recruited.

Regarding cell islets (we call them patches of confluent cells as islets may be confused with e.g. more structured Langerhans islets), there are hardly any PsVs at the basal membrane. We state on line 135: “Frequently, we observe patches of confluent cells which are common to HaCaT cells. Cells at the center of these patches are dismissed during imaging, because hardly any PsVs are bound to their basal membrane, indicating that PsVs do rather not reach this area by passive diffusion. Instead, we focus on isolated HaCaT cells or cells at the periphery of cell patches. At these cells, we find more PsVs per cell than one would expect from the employed ≈ 50 viral genome equivalents (vge) per cell, indicating that PsVs are unequally distributed between the cells.”

Is the difference between untreated and cytoD treated significant?

We stated in the Figure legend that the difference is not significant (the exact p value is p = 0.089). We now have revised the Figure (previously Supplementary Figure 1A and B, now Supplementary Figure 6A and B), showing the PsV density at the basal membrane over time, also for the experiment shown in Figure 6. The now revised Figure (Supplementary Figure 6A and B) is discussed together with the re-attachment experiment (Supplementary Figure 6C and D), in order to compare the PsV accessibility to the cell membrane with and without diffusion barrier. Please see our reply above (paragraph starting at line 447).
